# The association of low serum salivary and pancreatic amylases with the increased use of lipids as an energy source in non-obese healthy women

**DOI:** 10.1186/s13104-020-05078-2

**Published:** 2020-05-06

**Authors:** Kei Nakajima, Ryoko Higuchi, Taizo Iwane, Ayaka Iida

**Affiliations:** 1 School of Nutrition and Dietetics, Faculty of Health and Social Services, Kanagawa University of Human Services, 1-10-1 Heisei-cho, Yokosuka, Kanagawa 238-8522 Japan; 2 Department of Endocrinology and Diabetes, Saitama Medical Center, Saitama Medical University, 1981 Kamoda, Kawagoe, Saitama 350-8550 Japan; 3 Graduate School of Health Innovation, Kanagawa University of Human Services, Research Gate Building Tonomachi 2-A, 3-25-10 Tonomachi, Kawasaki, Kanagawa 210-0821 Japan

**Keywords:** Salivary, Pancreatic, Amylase, Ketones, Respiratory quotient, BMI, HbA1c

## Abstract

**Objective:**

It is unknown whether low serum levels of salivary and pancreatic amylases are associated with the high combustion of carbohydrates or lipids for energy. Elevated blood ketones and a low respiratory quotient (RQ) can reflect the preferential combustion of lipids relative to carbohydrates. Therefore, using the data from our previous study, we investigated if low levels of serum amylases were associated with a high serum ketone level and low RQ in 60 healthy non-obese young women aged 20–39 years old.

**Results:**

Serum ketones [3-hydroxybutyric acid (3-HBA) and acetoacetic acid (AA)] were inversely correlated with RQs, but not body mass index (BMI) or glycated haemoglobin (HbA1c) levels. Logistic regression analysis showed that high levels of serum ketones (3-HBA ≥ 24 μmol/L and AA ≥ 17 μmol/L) and a low RQ (< 0.766) were significantly associated with low serum salivary (< 60 U/L) and pancreatic (< 29 U/L) amylase levels, respectively. These associations were not altered by further adjustments for age, BMI, HbA1c, and estimated glomerular filtration rate. These results confirm the high combustion of lipids for energy in individuals with low serum amylase levels, suggesting a close relationship between circulating amylases and internal energy production.

## Introduction

We previously showed an association between low serum amylase and positive ketonuria assessed by dipstick urinalysis in a general population of Japanese adults who underwent a health-screening check-up. These results suggested a lower availability of carbohydrates for energy production in individuals with low serum amylase levels [1]. However, the determination by ketonuria using dipstick urinalysis may be inaccurate with dichotomized results: negative or positive responses [2, 3]. By contrast, serum ketones, such as 3-hydroxybutyric acid (3-HBA) and acetoacetic acid (AA) can reflect a more accurate condition related to the combustion of lipids caused by increased β-oxidation of fatty acids in the liver [4–6]. The level of respiratory quotient (RQ), which is the ratio of CO_2_ produced to O_2_ consumed, also reflects the combustion of macronutrients while food is being metabolized [7, 8]. In our recent study conducted for the purpose of elucidating association between salivary amylase gene and glucose metabolism in 60 healthy young Japanese women aged 20–39 years [9], we found no significant correlation of serum salivary and pancreatic amylase with serum ketones. However, these correlations were tested from the viewpoint of continuous variables. Additionally, these correlations were not evaluated controlling for confounding factors such as age, body mass index (BMI) and glycated haemoglobin (HbA_1c_). Although many clinical studies have shown that low serum amylase is significantly associated with diabetes and obesity [10, 11], underlying mechanism and potential treatments for such physiology (low serum amylase and diabetes) including diet therapy remain unknown. Therefore, to confirm and advance our previous findings [1, 9], we aimed to investigate the association of low serum salivary and pancreatic amylases with high serum ketones and a low RQ by conducting a sub-analysis of the data from our previous study that consisted of healthy non-obese young women [9].

## Main text

### Materials and methods

#### Study design and participants

We reanalysed the data from our previous study [9] that consisted of 60 healthy Japanese women aged 20–39 who were non-smokers, had a normal BMI (< 25.0 kg/m^2^), and had no history of metabolic disorders including diabetes and dyslipidemia.

#### Measurements

Anthropometric and laboratory measurements were obtained in the morning following an overnight fast. Biochemical parameters, including glycated haemoglobin (HbA_1c_), ketones (3-HBA and AA), and amylase (salivary and pancreatic), were measured using standard methods by SRL, Inc., a Japanese clinical laboratory test company. HbA1c (Japan Diabetes Society) was converted to HbA1c (National Glycohemoglobin Standardization Program) [12]. RQs at rest for five minutes were measured using an AR-1 portable gas monitor (ARCO SYSTEM Inc, Japan). The degree of estimated glomerular filtration rate (eGFR) was also considered as a confounding factor because kidneys play a major role in eliminating circulating amylase [13, 14]. eGFR was calculated for Japanese female subjects using the following equation [15].

$${\text{eGFR }}\left( {{\text{ml}}/{ \hbox{min} }/ 1. 7 3 {\text{m}}^{ 2} } \right)\, = \, 1 9 4\, \times \,{\text{serum creatinine }}\left( {{\text{mg}}/{\text{dl}}} \right)^{ - 1.0 9 4} \, \times \,{\text{age}}^{ - 0. 2 8 7} \, \times \,0. 7 3 9.$$

### Statistical analysis

Because the distributions of serum ketones and RQs were expected to be skewed, the correlations of these were tested by Spearman’s rank correlation. Since the median levels of 3-HBA, AA, serum salivary amylase, and serum pancreatic amylase were 24 µmol/L, 17 µmol/L, 60 U/L, and 29 U/L, respectively, high levels of serum 3-HBA and AA and low levels of serum salivary and pancreatic amylases were determined as ≥ 24 µmol/L, ≥ 17 µmol/L, < 60 U/L, and < 29 U/L, respectively, in this study. The proportion of serum salivary amylase in total (salivary + pancreatic) serum amylase was also considered. Low proportion of serum salivary amylase was determined as < 66%, which was the mean of proportion of serum salivary amylase.

The RQ is usually divided into tertile in terms of the combustion of the three macronutrients (0.9–1.0 for carbohydrates, 0.8–0.9 for proteins or mixture, and 0.7–0.8 for lipids) [8]. The lowest tertile of the RQ equivalent to lipid combustion was 0.766 in this study. Therefore, to investigate the combustion of lipids, a low RQ was determined as < 0.766. Logistic regression analysis was used to test the association of low serum salivary and pancreatic amylases with low serum ketones and RQs considering confounding factors (age, BMI, HbA1c, and eGFR). Low proportion of serum salivary amylase was also tested instead of low salivary amylase. Statistical analysis was performed using SAS Enterprise Guide (SAS-EG 7.1) in SAS version 9.4 (SAS Institute, Cary, NC, USA). A p value < 0.05 was considered to indicate statistical significance.

## Results

**Fig. 1:**
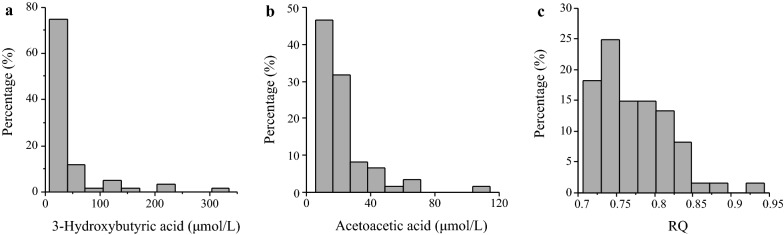
Distributions of 3-Hydroxybutyric acid, acetoacetic acid, and RQ. **a** 3-Hydroxybutyric acid; **b** acetoacetic acid; **c** RQ

The characteristics of the participants were reported in our previous study [9], which indicated that the mean of each parameter was within the normal range. Figure 1 shows the distributions of serum ketones, which were highly skewed to the lowest level, whereas the distribution of the RQ was mildly skewed to a low level. The distributions of serum amylases are shown in Additional file 1: Fig S1. An almost normal distribution was observed in the concentration of serum salivary amylase, whereas the distribution of the serum pancreatic amylase was unclear.

Although data is not shown, serum ketones were significantly correlated with RQ (r = − 0.45, p = 0.0005 for 3-HBA and r = − 0.36, p = 0.006 for AA) but not BMI (r = − 0.06, p = 0.63 for 3-HBA and r = − 0.10, p = 0.45 for AA) or HbA1c (r = − 0.10, p = 0.46 for 3-HBA and r = − 0.07, p = 0.60 for AA). In addition, no significant correlation was observed between RQ and BMI (r = − 0.09, p = 0.50) or between RQ and HbA1c (r = − 0.12, p = 0.35).

Logistic regression analysis showed that low serum salivary amylase (< 60 U/L) was significantly associated with high levels of serum 3-HBA (≥ 24 μmol/L) and AA (≥ 17 μmol/L), but not with a low RQ (< 0.766) (Table 1), which were not altered by the further adjustment for age, BMI, HbA1c, and eGFR (Model 3). Table 2 shows the associations of low serum pancreatic amylase (< 29 U/L) with high serum ketones and a low RQ. Low serum pancreatic amylase was significantly associated with a low RQ, but not with high levels of serum 3-HBA or AA (Table 2). These associations were not altered by further adjustment for confounders (Model 3). No significant association was observed between low proportion of serum salivary amylase (< 66%) and high levels of serum 3-HBA, AA, and low RQ, regardless of the adjustment for confounders (data not shown).

**Table 1:** Odds ratios of low salivary amylase for high serum ketones and low RQ

	Normal-high serum salivary amylase N = 27	Low serum salivary amylase N = 33	P values
High serum 3-HBA, n (%)	10 (32.3)	21 (67.7)	
	Odds ratios (95% ICs)		
Model 1	1 (reference)	2.98 (1.04–8.55)	0.04
Model 2	1 (reference)	3.40 (1.12–10.3)	0.03
Model 3	1 (reference)	4.40 (1.32–14.7)	0.02

High serum AA, n (%)	9 (33.3)	23 (69.7)	P values
	Odds ratios (95% ICs)		
Model 1	1 (reference)	4.60 (1.54–13.7)	0.006
Model 2	1 (reference)	5.42 (1.70–17.3)	0.004
Model 3	1 (reference)	6.64 (1.91–23.1)	0.003

Low RQ, n (%)	7 (25.9)	13 (39.4)	P values
	Odds ratios (95% ICs)		
Model 1	1 (reference)	1.86 (0.61–5.63)	0.27
Model 2	1 (reference)	1.96 (0.62–6.21)	0.25
Model 3	1 (reference)	1.87 (0.56–6.20)	0.31

Model 1: unadjusted

Model 2: adjusted for age and BMI as continuous variables

Model 3: model 2 with additional adjustments for HbA1c and eGFR as continuous variables

Low serum salivary amylase: < 60 U/L (vs. normal-high serum salivary amylase, ≥ 60 U/L)

High serum 3-Hydroxybutyric acid ≥ 24 μmol/L (vs. normal serum 3-Hydroxybutyric acid, < 24 μmol/L)

High serum acetoacetic acid ≥ 17 μmol/L (vs. normal serum acetoacetic acid, < 17 μmol/L)

Low RQ: < 0.766 (vs. normal-high RQ, ≥ 0.766)

AA, acetoacetic acid; BMI, body mass index; eGFR, estimated glomerular filtration rate; 3-HBA, 3-Hydroxybutyric acid; RQ, respiratory quotient

**Table 2:** Odds ratios of low pancreas amylase for high serum ketones and low RQ

	Normal-high serum pancreas amylase N = 31	Low serum pancreas amylase N = 29	P values
High serum 3-HBA, n (%)	14 (45.2)	17 (58.6)	
	Odds ratios (95% ICs)		
Model 1	1 (reference)	1.72 (0.62–4.79)	0.30
Model 2	1 (reference)	2.13 (0.73–6.26)	0.17
Model 3	1 (reference)	2.41 (0.79–7.36)	0.12

High serum AA, n (%)	14 (45.2)	18 (62.1)	P values
	Odds ratios (95% ICs)		
Model 1	1 (reference)	1.99 (0.71–5.57)	0.19
Model 2	1 (reference)	2.23 (0.77–6.49)	0.14
Model 3	1 (reference)	2.20 (0.73–6.56)	0.16

Low RQ, n (%)	6 (19.4)	14 (48.3)	P values
	Odds ratios (95% ICs)		
Model 1	1 (reference)	3.89 (1.23–12.3)	0.02
Model 2	1 (reference)	3.37 (1.04–11.0)	0.04
Model 3	1 (reference)	5.21 (1.41–19.2)	0.01

Model 1: unadjusted

Model 2: adjusted for age and BMI as continuous variables

Model 3: model 2 with additional adjustments for HbA1c and eGFR as continuous variables

Low serum pancreatic amylase: < 29 U/L (vs. normal-high serum salivary amylase, ≥ 29 U/L)

High serum 3-Hydroxybutyric acid ≥ 24 μmol/L (vs. normal serum 3-Hydroxybutyric acid, < 24 μmol/L)

High serum acetoacetic acid ≥ 17 μmol/L (vs. normal serum acetoacetic acid, < 17 μmol/L)

Low RQ: < 0.766 U/L (vs. normal-high RQ, ≥ 0.766 U/L)

AA, acetoacetic acid; BMI, body mass index; eGFR, estimated glomerular filtration rate; 3-HBA, 3-Hydroxybutyric acid; RQ, respiratory quotient

## Discussion

In the current study, we found significant associations between low serum salivary amylase and high serum ketones, and between low serum pancreatic amylase and low RQs in healthy subjects. These associations were independent of relevant confounders including age, BMI, HbA1c, and eGFR. However, the proportion of salivary to total amylase was unlikely to relate with high serum ketones and low RQ, although the proportion of salivary amylase was correlated with the level of blood glucose at early time point after starch loading in our previous study [9].

Because subjects in this study are healthy non-obese young female non-smokers, current results indicate fundamental relationship among serum salivary and pancreatic amylase and metabolic indices such as blood ketones and RQ. As both high serum ketones and low RQs reflect the high combustion of lipids compared with carbohydrates [4–6], our current findings suggest that individuals with low serum salivary and pancreatic amylases may obtain their energy predominantly by lipid combustion (fatty acid oxidation), which is consistent with our previous study that showed an association between low total serum amylase and ketonuria in a heterogeneous population with a broad range of age (25–79 years) [1].

Notably, in our previous study [1], no significant inverse correlation between serum ketones and serum salivary and pancreatic amylases, which were assessed as continuous variables, was observed. However, significant associations between low serum salivary amylase and high serum ketones were observed in this study. This discrepancy may depend on the difference in statistical methods between nonparametric correlation tests in the previous study and logistic regression analysis in the current study, likely because serum ketones were highly skewed almost to an undetectable level (Fig. 1a, b). In contrast, the previous study showed a positive correlation between serum pancreatic amylase and RQ, which is consistent with the observed association between low pancreatic amylase and low RQs in this study. The degree of skewness of RQ values is mild compared with those of serum ketones (Fig. 1c), which may contribute to the similar RQ results of this study and the previous study.

It has been shown that reduced rates of fat oxidation, namely, high RQ, may contribute to the predisposition to obesity or weight gain [16–18]. However, the predisposition to fat accumulation is associated with high tissue sensitivity to insulin [19, 20]. Schutz showed in his study [16] that high RQ, low fat oxidation, and high insulin sensitivity (predictors) were observed in the dynamic phase, whereas low RQ, high fat oxidation, and insulin resistance (outcomes) were observed in the static phase (compensated state). In line with this, we have considered potential underlying mechanism for the current findings (Additional file 2: Figure S2), although this study is a cross-sectional study in nature. Baseline individual levels of serum amylases, which are genetically determined in most cases, are likely to be influenced by other factors including obesity and insulin resistance, eventually resulting in alternation of energy metabolism. The subjects with low serum salivary or pancreatic amylases observed in this study may be at the static phase or feedback phase because low RQ and high serum ketones (high fat oxidation) were associated with low serum pancreatic and salivary amylase, respectively.

Although high levels of ketones often reflect a deficiency in insulin secretion in diabetic patients [2–4], no subjects in this study had diabetes or impaired glucose metabolism. By contrast, the ketogenic diet, which involves carbohydrate restriction, frequently causes elevated serum ketones, even in healthy individuals [3, 4]. Because current subjects were young women aged 20-39 years, a proportion of the subjects may have conducted such a diet during this study, which may have contributed to the high levels of serum ketones, especially in the fasted state in the morning. This issue deserves further study.

Although current study indicates that fatty acids may be predominantly used as an energy source in individuals with low serum amylase, it is unclear if a diet rich in lipids is suitable for individuals with low serum amylase. Taken together, our studies suggest that a close relationship may exist between serum amylase and the specific type of macronutrient combustion used for energy.

While increases in serum amylase occur because of leakage from salivary glands and the pancreas, the clinical relevance of this remains unknown. Circulating amylases might just be a marker of leaks or damage, albeit several investigators have suggested a feedback system between serum amylase and insulin action [10, 11, 21]. Insulin resistance may downregulate the production of amylase [21], possibly for the purpose of reducing absorption of glucose digested from starch. On the contrary, high levels of serum amylase can reduce the secretion of insulin in the pancreas [21]. However, it is unknown whether this plausible feedback system is also applicable in the salivary gland.

In conclusion, the current results obtained from the reanalysis of our previous study confirm the high combustion of lipids for energy in individuals with low serum amylases, suggesting a close relationship between circulating amylases and internal energy production.

## Limitations

First, although low serum amylases may be caused by insulin resistance and reduced insulin secretion [10, 21], insulin levels were not measured in this study, and therefore, precise underlying mechanism remains to be elucidated. Second, this study was conducted on a relatively small sample of 60 Japanese women, which can influence the reliability of our results. However, the enrolled subjects in this study were considered as homogeneous in nature because they were all young healthy non-obese non-smokers without metabolic abnormality. Therefore, current results may reflect a fundamental physiological relationship between serum amylases and metabolic indices, regardless of small sample size. On the contrary, the present results may not be applicable to other populations, such as those in western countries, who may have lower blood amylases [22] than the current subjects.

## Supplementary information

**Additional file 1: Figure S1.** Distributions of serum amylases. A, serum salivary amylase; B, serum pancreatic amylase; C, serum total amylase.

**Additional file 2: Figure S2.** Potential underlying mechanism between serum amylases and metabolic indices. *Baseline individual levels of serum amylases, which are genetically determined in most cases.

## Data Availability

A confidentiality agreement with participants prevents us from sharing the data.

## Abbreviations

RQ: Respiratory quotient
3-HBA: 3-hydroxybutyric acid
AA: Acetoacetic acid
BMI: Body mass index
HbA_1c_: Glycated haemoglobin
eGFR: Estimated glomerular filtration rate
