# Supplementary material for: The association of low serum salivary and pancreatic amylases with the increased use of lipids as an energy source in non-obese healthy women
Source: BMC Res Notes. 2020 May 6;13:237. doi: 10.1186/s13104-020-05078-2 (PMC7201991; doi:10.1186/s13104-020-05078-2)
Supplement: Supplementary file 1 — Additional file 1: Figure S1. Distributions of serum amylases. A, serum salivary amylase; B, serum pancreatic amylase; C, serum total amylase. [file 13104_2020_5078_MOESM1_ESM.docx]

Fig S1

0

5

10

15

20

10

20

30

40

50

60

Serum pancreatic amylase (U/L)

Percentage (%)

0

5

10

15

20

25

0

20

40

60

80

100

120

Serum salivary amylase (U/L)

Percentage (%)

0

5

10

15

20

20

40

60

80

100

120

140

160

Serum total amylase (U/L)

Percentage (%)

**C**

**B**

**A**
