# Supplementary material for: The association of low serum salivary and pancreatic amylases with the increased use of lipids as an energy source in non-obese healthy women
Source: BMC Res Notes. 2020 May 6;13:237. doi: 10.1186/s13104-020-05078-2 (PMC7201991; doi:10.1186/s13104-020-05078-2)
Supplement: Supplementary file 2 — Additional file 2: Figure S2. Potential underlying mechanism between serum amylases and metabolic indices. *Baseline individual levels of serum amylases, which are genetically determined in most cases. [file 13104_2020_5078_MOESM2_ESM.docx]

Figure S2

Improvement of

insulin resistance

Low salivary amylase

Low pancreatic amylase

Baseline levels of serum amylases*

Reduced absorption of carbohydrate

Persistent high carbohydrate intake

or

High energy intake

Obesity

Insulin resistance

High insulin

sensitivity

Amelioration of

Obesity

Fat accumulation ↑

Carbohydrate oxidation ↑

(High RQ, Low serum ketones)

Fat oxidation ↑

Carbohydrate oxidation ↓

(Low RQ, High serum ketones)

Static phase

(compensate or feedback phase)

Dynamic phase

Improvement of

insulin resistance
